# Feature-Selection and Mutual-Clustering Approaches to Improve DoS Detection and Maintain WSNs’ Lifetime

**DOI:** 10.3390/s21144821

**Published:** 2021-07-15

**Authors:** Rami Ahmad, Raniyah Wazirali, Qusay Bsoul, Tarik Abu-Ain, Waleed Abu-Ain

**Affiliations:** 1 The School of Information Technology, Sebha University, Sebha 71, Libya; r_a_sh2001@yahoo.com; 2 College of Computing and Informatics, Saudi Electronic University, Riyadh 11673, Saudi Arabia; t.aboain@seu.edu.sa; 3 Faculty of Science and Information Technology, Irbid National University, Irbid 21110, Jordan; q.bsoul@inu.edu.jo; 4 College of Community, Taibah University, Badr 46354, Saudi Arabia; wabuain@taibahu.edu.sa

**Keywords:** IDS, machine learning, DoS, WSN security, feature selection, LEACH

## Abstract

Wireless Sensor Networks (WSNs) continue to face two major challenges: energy and security. As a consequence, one of the WSN-related security tasks is to protect them from Denial of Service (DoS) and Distributed DoS (DDoS) attacks. Machine learning-based systems are the only viable option for these types of attacks, as traditional packet deep scan systems depend on open field inspection in transport layer security packets and the open field encryption trend. Moreover, network data traffic will become more complex due to increases in the amount of data transmitted between WSN nodes as a result of increasing usage in the future. Therefore, there is a need to use feature selection techniques with machine learning in order to determine which data in the DoS detection process are most important. This paper examined techniques for improving DoS anomalies detection along with power reservation in WSNs to balance them. A new clustering technique was introduced, called the CH_Rotations algorithm, to improve anomaly detection efficiency over a WSN’s lifetime. Furthermore, the use of feature selection techniques with machine learning algorithms in examining WSN node traffic and the effect of these techniques on the lifetime of WSNs was evaluated. The evaluation results showed that the Water Cycle (WC) feature selection displayed the best average performance accuracy of 2%, 5%, 3%, and 3% greater than Particle Swarm Optimization (PSO), Simulated Annealing (SA), Harmony Search (HS), and Genetic Algorithm (GA), respectively. Moreover, the WC with Decision Tree (DT) classifier showed 100% accuracy with only one feature. In addition, the CH_Rotations algorithm improved network lifetime by 30% compared to the standard LEACH protocol. Network lifetime using the WC + DT technique was reduced by 5% compared to other WC + DT-free scenarios.

## 1. Introduction

Wireless network technology is at the heart of the growth of the Internet of Things (IoT). This is because wireless networks are critical for transmitting interactive data from devices to humans, as well as between devices [1]. These devices are part of automation and control systems, embedded systems, Wireless Sensor Networks (WSNs), and other systems that exchange data in a variety of environments without requiring human intervention. Most applications that use these devices are made up of perception, network, and application layers [2]. The application and network layers are mostly executed in high-powered devices, while the perception layer is mostly executed in low-powered devices to keep them running as long as possible, particularly when using systems with limited battery life. Since perception devices depend on public wireless networks, the perception layer is considered one of the most sensitive topics in need of attention, particularly to protection against attack [3]. Accreditation within this layer contributes to many issues in WSN architecture. One of these issues applies to security, privacy, and availability within the perception layer [4]. Attackers can listen in on radio transmissions, send fake messages over communication channels, and alter received data packets [5,6]. Moreover, they can use compromised WSN nodes with similar hardware resources to legitimate network nodes [7]. In addition, an attacker might be capable of stopping WSN node services by using various attacks such as sinkhole, wormhole, hello flood, Sybil, and Denial of Service (DoS) [8].

DoS and Distributed DoS (DDoS) attacks are one of the most common and dangerous threats to WSN security, as they occur when several compromised WSN nodes are infected by malicious WSN nodes at the same time under the control of a single attacker by overwhelming the target WSN nodes with bogus requests. This depletes their resources and forces them to refuse services to legitimate WSN nodes [9]. Therefore, in this paper, we will focus on slowing down DoS and DDoS attacks in WSNs with minimum power consumption and good detection accuracy.

Due to the inability to prevent or completely stop such types of attacks, Intrusion Detection Systems (IDS) are used to discover suspicious or abnormal activities and alert the WSN nodes [10]. Signature-based and anomaly-based intrusion detection are the two types of intrusion detection. In an anomaly pattern, the system must regularly track network access and compare ongoing WSN operations to normal traffic patterns [10,11]. However, the anomaly detection technique needs to examine and analyze each transmission packet in detail. Thus, it consumes energy and CPU, which are weaknesses of WSN nodes. As a result, a variety of techniques have been used to boost DoS detection efficiency using both active and passive methods. Supervised machine learning algorithms are one such technique used to predict and classify DoS and DDoS attacks [12,13,14,15]. Decision Tree (DT), Support Vector Machine (SVM), K-Nearest Neighbours (KNN), Deep-Learning (DL) classifier, and Naive Bayes (NB) are common algorithms for this purpose [16]. The authors in [1,2,9,12,14,17,18] used various deep learning mechanisms, and their results in terms of detection precision, mean squared error, and sensitivity were satisfactory, but none of them addressed the impact of their proposals on WSNs, such as node power consumption and network lifetime. The problem with these techniques is the amount of data they require for the training and testing process, and WSN nodes’ inability to manage such a huge number of dimension records. Therefore, another technology can be used in conjunction with machine learning classifiers is a feature selection algorithm. This is used to determine which data features are most important in the IDS process [19], and helps in understanding data, minimizing computation requirements, and improving prediction performance. Examples of feature selection technologies are Water Cycle (WC) [20], Particle Swarm Optimization (PSO) [21], Simulated Annealing (SA) [22], Harmony Search (HS) [19], and Genetic Algorithm (GA) [23].

Since the main objective of this paper is to find the best solution to protect the perception layer from DoS attacks while taking into account the capabilities of these devices, we will analyze the effects of various machine learning algorithms along with different feature selection techniques in order to balance their performance accuracy for DoS detection with their consumption energy. As some studies, such as [24,25], have shown that machine learning techniques (logistic regression, SVM, and DT) are more appropriate for real-world deployment of wireless devices than a deep learning mechanism, machine learning techniques are favored. That is due to the need for a significant amount of training data for deep learning algorithms in order to provide high-accuracy classification performance. On the other hand, the IDS technique in WSNs must be compatible with their protocols. In WSNs there are different routing protocols that are used to transmit data packets to the Access Point (AP), such as Low Power and Lossy Networks (RPL) in 6LoWPAN [26], Adhoc On Demand Distance Vector (AODV) routing in ZigBee [27], and Low Energy Aware Cluster Hierarchy (LEACH) [28]. LEACH is one of the most widely used hierarchical routing protocols due to its limited power consumption, and is used in this study due to the fact that the WSN-Data Set [29] on which we rely for analysis was collected according to this protocol. It aims to boost energy efficiency by using a rotation-based Cluster Head (CH) selection process with a random number. However, the reliance on randomness in rotation-based CH selection is considered a weakness in the LEACH protocol, and various studies [30,31,32,33,34] have improved its performance. It remains possible to improve the protocol further in terms of increasing its efficiency in selecting the appropriate CH in each loop, and increasing the network lifetime. By summarizing previous studies and applications, we see that there has been an increase in the use of WSNs recently, that these devices play multiple roles, and the biggest challenges for WSNs remain security and energy. To overcome these challenges, several solutions that discuss data security performance problems have been proposed [10,11,12,13,15,35,36] that do not consider the impact on wireless power consumption. Moreover, other studies have discussed the concept of energy conservation from a data security-independent perspective [30,31,32,33,34]. Therefore, in this study, we will create a complete picture that combines both elements (security and energy), by determining how best to raise the level of security in WSNs and protect them from DoS attacks with minimal energy consumption. We modified the cluster protocol to increase network performance efficiency while saving energy and increasing DoS detection. Moreover, in certain situations, WSN nodes must be used in sensitive areas and we cannot power them up regularly; thus, we sought to save as much energy as possible while preserving security. In this work, we will contribute to improving DoS detection and reduce power consumption in WSNs. The following are the main contributions of this work:

1. We modified the LEACH protocol to reduce randomness in determining the CH nodes by adding other factors such as node residual power, distance between nodes, and distance to AP in order to increase efficiency and extend the lifetime of the WSN.
2. We analyzed the effect of feature selection techniques along with machine learning algorithms on the accuracy of DoS detection.
3. We studied the effect of this modified LEACH protocol on the best-performing technique in terms of network lifetime. This is in contrast to related studies that have improved the accuracy of DoS detection over WSN without analyzing its actual effect on sensors.

The rest of our paper is set out as follows. The second section covers related work in WSNs, DoS attacks, feature selection techniques, and machine learning algorithms. The methodology, environmental development, cluster management, feature selection machine learning test, and decision-making are all covered in Section 3. Data collection and arrangement are covered in Section 4. Section 5 delves into the implementation and assessment of complexity analysis in feature selection and machine learning techniques, as well as the lifetime of WSNs. In Section 6, the paper’s conclusions and directions for future work are presented.

## 2. Background and Related Works

In this section we will provide a literature review and technical background concerning WSNs, clustering efficiency and intrusion detection in these networks, and machine learning algorithms in intrusion detection.

### 2.1. LEACH Protocol Energy Efficiency in WSNs

A WSN is a radio access spectrum that uses a frequency of 2.4 GHz which is designed to work in low-power, low-range, and low-processor circumstances. Every WSN has different WSN nodes that communicate with each other and their Access Point (AP). Data are transferred from WSN node to AP through different routing protocols. One of these protocols, LEACH, is used to reduce the power consumption of the WSN nodes, which have limited power capacity. The key concept of this protocol is to spread the energy load of the entire network equally to each WSN node by selecting WSN CH nodes at random in each loop. Due to the CH-shifting technique used in the LEACH protocol, all WSN nodes are believed to have a similar survival time [37]. The CH selection process in LEACH takes place in two phases in each loop: CH establishment and steady-state [38]. In the CH establishment phase, each WSN node generates a random value between 0 and 1, and then starts computing the threshold formula *Thrd(n).* Subsequently, in each WSN node if the random chosen value is less than *Thrd(n),* it becomes a WSN CH node and it will send request messages to neighboring ordinary WSN nodes. The formula *Thrd (n)* is illustrated in (1)

(1) $$Thrd\left(n\right)=\{\begin{array}{c} \frac{1\;}{1-p\left(r\;mod\;\frac{1}{p}\right)\;}\;\;⩝n\;\in G\\ 0\;\;\;\;\;\;\;\;\;otherwise \end{array}\;$$

where *n* is the WSN node, *p* is the probability that the *n* becomes CH, *r* is the current loop number, and G represents the group of WSN nodes that have not joined in the previous CH selection loops (1/*p*).

**Figure 1:**
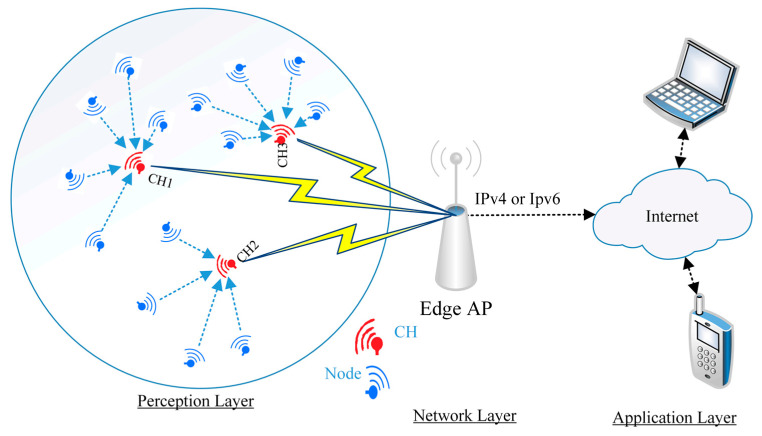
The architecture of the LEACH protocol in WSNs.

We conclude from this that a WSN node that is chosen as CH for the *r* loop will not participate in the loops following *r*. Thus, all WSN nodes have the same chance to be CH nodes. In the steady-state phase, the ordinary WSN nodes in each CH transmit the data to their cluster WSN node by using the Time-Division Multiple Access (TDMA) schedule. The LEACH protocol architecture is illustrated in Figure 1.

As shown in Figure 1, the WSNs are responsible for drawing the network topology and routing table in the perception layer using different protocols [39] as explained earlier. Each WSN node associates with the set of its neighboring peer WSN nodes, after which the WSN node starts collecting data from different locations and forwarding the data to the network layer (edge-AP). Therefore, much research has been done to improve the LEACH protocol in the perception layer and thus reduce the WSN nodes’ consumption power. The authors in [32] altered the LEACH protocol through using a number of WSN node links and factoring in the cost of the path for choosing CH nodes rather than a random selection technique. In [33], the authors studied the effect of shortening the distance between ordinary WSN nodes and CH nodes from one side and the effect of shortening the distance between CH nodes and AP from the other side on network lifetime. The result showed a reduction in power consumption. The authors in [40] optimized a new routing protocol through employing three complementary steps in each CH selection loop. First, it determined the optimal CH numbers used in WSNs, then it created a Voronoi diagram in between neighboring WSN nodes to determine CH nodes. Finally, it used an ant colony algorithm to optimize the multi-hop routing protocol. However, through using all of these steps in every setting, the CH loop exhausted the network power and CPU. In addition, [31] created an algorithm called a standardized experimental bat has been proposed to improve CH selection in each loop. This algorithm worked on the basis of a search balance between local and global WSN nodes in a WSN mobile node environment. To examine the feasibility of the use of Global Positioning System (GPS), the authors in [30] divided the area of the network belonging to the coverage area of the AP into different zones. In each CH determination loop, one WSN node was identified as a CH node in each zone, and the role was rotated between WSN nodes that occurred in the same zone. However, this technology does not deal with a large number of WSN nodes [41]. Therefore, the LEACH protocol remains amenable to improvement through increased efficiency in selecting the appropriate CH in each loop, and that will be one of the contributions of this paper.

A User Datagram Protocol (UDP) is used as the data transmission protocol in WSNs to reduce the packet’s clustering complexity and reduce CPU overhead. Moreover, to secure data transmission over UDP, the Datagram Transport Layer Security (DTLS) protocol is used on top of UDP [42]. However, these WSN nodes are designed to operate in various untrusted surroundings, which are not periodically monitored. This makes WSN nodes vulnerable to various security attacks, especially if they are related to important and sensitive data [43]. Moreover, with regard to WSN nodes’ operational boundaries in CPU power and energy [44], it is sometimes difficult to provide a charger for them in these conditions.

### 2.2. DoS in WSNs

As mentioned previously, the main objective of the DoS or DDoS attack is to affect the network’s availability by disrupting services and network performance. Therefore, the effect of this type of attack varies depending on the network layer stack [5]. Since wireless sensor networks have stacks of five network layers, each layer is vulnerable to different types of attacks [10,45]. The attached Table 1 shows each layer and type of DDoS attack that can be represented.

Blackhole and grayhole DDoS attacks affect the routing protocol in layer three by declaring the attacker node itself as the cluster head, and we will discuss the cluster head functionality in detail in the clustering management subsection. By comparison, a flooding attack affects WSN availability by sending a large number of advertising messages to cluster heads. In scheduling attacks, the perception and MAC layers’ activity is targeted by changing the broadcast channel schedule to a unicast channel schedule. This change leads to packet collision and later data loss [29].

Several researchers have attempted to mitigate DDoS or DoS in WSNs. The Message Authentication System (MAS) algorithm was used by the authors in [7] to locate and delete DoS attacks. The proposal divides WSN nodes into different clusters, with each cluster head using the MAS method to separate legitimate traffic from phishing scams. The authors in [20,46] improved the k-means clustering scheme for finding DDoS and misdirection attacks. To detect attacks in a home WSN, the authors of [47] used user-behaviors learning analysis. In [18], the authors used Restricted Boltzmann Machine-based Clustered IDS (RBC-IDS), a deep learning-based technique for tracking sensitive infrastructure utilizing three hidden layers for potential intruders. Authors in [48] used a genetic algorithm combined with a Multi-Layer Perceptron algorithm to improve detection methods. In [49], the authors presented a novel swarm optimization algorithm for clustering WSN nodes and then used a VSM classifier to detect DoS attacks in each cluster. However, rather than anti-attack initiatives, the focus of this study will be on analyzing DoS attack detection efficiency. In this work we will look at four types of DoS attacks, namely Blackhole, Grayhole, Flooding, and Scheduling.

### 2.3. Feature Selection and Machine Learning in Intrusion Detection Approach

Feature subset selection is a common problem in network detection [23] due to the high dimensionality of features of the sensor. Therefore, the development of new approaches to handle feature selection is still an active area of research, particularly for feature identification. The purpose of feature selection is to improve performance in areas such as accuracy, data visualization and simplification for model selection, and dimensionality reduction to remove noise and irrelevant features [50]. The selection of the features and distribution of the data have a high impact on the performance of classifier algorithms. These tend to obtain local minima rather than the global minimum. The obtained results are often very good, especially when the initial features are fairly far apart. This is because the algorithm can usually distinguish the main category or class in a given data set. Moreover, the classifier algorithm’s main process and the quality of feature identification are both affected by the initial feature selection. Thus, the initial features can enhance the quality of the results [51]. There are two methods of feature selection: filter and wrapper. The filter method ignores feature dependencies [50,51]. The wrapper method uses optimization in feature selection and thus can provide a good initial choice of feature and perform better as features are refined and the best feature selection is found [50].

During the past two decades, a large number of classical optimization techniques have been developed to improve function, including the bat algorithm, biogeography-based optimization, bacterial research optimization, modified Lagrange approach, synthetic physics improvement, artificial plant improvement algorithm, generative models [52], PSO, GA, and cuckoo search. Nature-inspired heuristic algorithms have been used extensively, and more recently, the water cycle (WC) method [53], which is influenced by the behavior of rivers and seas in nature, has been used. The authors in [54] employed ant colony optimization as a feature selection method for classifiers and used the absolute value [55] as the similarity to optimize between features. The efficiency and effectiveness of the ant colony optimization were better than those of other feature selection methods used as classifiers. Abualigah et al. (2016) [56] employed a GA for feature selection and used the mean absolute difference as the similarity between features. They compared their proposed method with those of other feature selection methods and showed that their proposed method increased feature detection performance. Subsequently, Abualigah et al. [57] employed Particle Swarm Optimization (PSO) for feature selection. Their results showed that their proposed feature selection method outperformed other feature selection methods such as genetic and harmony search algorithms.

Additionally, feature selection techniques can be combined with machine learning algorithms to enhance the accuracy of results. Machine learning is a method that improves or learns from an interpretation or experience without requiring manual configuration. It can be divided into supervised and unsupervised learning. Classification and regression are two types of supervised learning. Statistical (SVM and Bayesian), logic-based (DT), instance-based (KNN), perceptron-based (deep learning (Recurrent Neural Networks (RNN), Long Short Term Memory (LSTM), Convolutional Neural Networks (CNN)) and Multi-Layer Perceptron (MLP) learning are the different types of classification [58]. The primary goal of this learning methodology is to develop a model that defines the relationships and dependencies between input features and predicted objective outcomes [16]. As a result, supervised learning can be used to solve real world problems in WSNs, including fault and anomaly detection. Table 2 shows the different types of detection methods and machine learning strategies used in attack detection for WSNs.

In this paper we will analyze WSNs’ traffic based on different feature selection techniques combined with machine learning classifications to see how they perform in detecting DoS attacks.

## 3. Proposed Methodology

**Figure 2:**
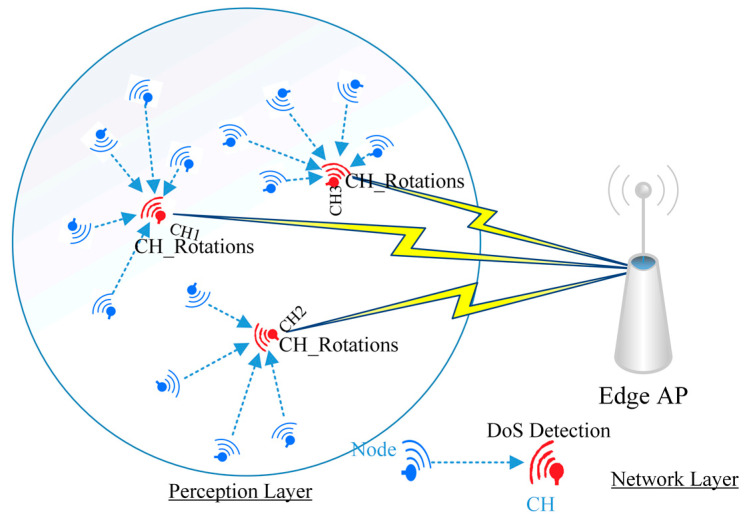
The structure of the general proposal model.

The main objective in this paper is to enhance the detection of anomalies in DoS with the lowest possible power consumption. First, we improve network performance through the use of the cluster protocol. Next, we combine lightweight feature selection with machine learning technologies to minimize DoS detection power cost and raise detection accuracy. Therefore, the proposal environment consists of three processes. The first is to aggregate WSN nodes into multiple clusters, each cluster having a CH in the WSN nodes environment. The LEACH standard protocol is updated with additional parameters to improve its performance, and the protocol generated from the modifications denoted CH_Rotations. In the following process, feature selection and machine learning testing approaches are used to analyze traffic in each CH node to distinguish between normal and abnormal incoming packets. The feature selection technique is used to detect the most important data features and also to reduce mathematical operations during each packet inspection at each WSN CH node. In the last process, CH makes a decision based on the results of the second process. The structure of the general proposal model is illustrated in Figure 2, and each of the processes will be discussed in the following subsections.

### 3.1. Clustering Management

The LEACH protocol increases power efficiency in WSNs by implementing CH_Rotations selection in each loop. However, the standard LEACH has various limitations regarding random CH selection, CH location relative to AP consideration, CH location relative to its ordinary nodes location, the number of ordinary nodes in each CH, and the number of CHs in each loop [38]. Therefore, we update the LEACH protocol to enhance its performance, as the distance between WSN nodes themselves and between them and the AP has a strong effect on power consumption during the communication process. The average distance between neighbour WSN nodes (*β*), the closest distance (*d*) to the AP, and the WSN node energy (*E*) are considered along with the LEACH parameters in the establishment phase to select the most suitable CH. Moreover, a new CH node can be selected in advance for the next loop, unlike in the standard protocol. The steady-state will be the same as the standard LEACH protocol. These alteration processes are depicted as follows:

1. Our work’s computing radio energy (*E*) model is based on [60] for each WSN node. The primary source of power usage is correspondence between WSN nodes, so power consumption is proportional to the distance between the transmitter and the receiver. The transmitter consumes power to operate the electrical transmission circuit and amplification, while the receiver consumes power to operate the cellular electronics of this embodiment. The power law of the distance between the transmitter and receiver can be used to shape the propagation of electromagnetic waves. As a result, the transmitter circuit uses P_TX-elec_ in proportion to the message transmitting size (*q*-bit) in terms of distance (*d*). The transmitter uses P_TX-amp_ to amplify the signal in order to produce a reasonable signal-to-noise ratio. The radio model’s cumulative power spent to transmit *q*-bit over distance *d(*P_TX_*)* is proposed to be (2):
  (2) $$P_{\mathrm{TX}}\left(q,d\right)=P_{\mathrm{TX}-\mathrm{elec}}\left(q\right){*P}_{\mathrm{TX}-\mathrm{amp}}\left(q,d\right)\;$$
  where P_TX-amp_ is equivalent to either β_fsm_ in a free space model or to β_trm_ in two-ray ground propagation models depending on distance between transmitter and receiver. Furthermore, a WSN node is in charge of transmitting data messages to other WSN nodes. As a consequence, WSN nodes will accept messages from other WSN nodes. Equation (3) can be used to measure the power needed to receive the *q*-bit message E_RX_:
  (3) $$E_{\mathrm{RX}}\left(q\right)=E_{\mathrm{RX}-\mathrm{elec}}\left(q\right)=E_{\mathrm{elec}}\times q\;$$
  where E_elec_ is the power absorbed by the transmitter and receiver per bit in nJ/bit, E_RX-elec_ is the power dissipated by the receiver during q-bits reception, and q is a bit-message.
2. The threshold energy (E_T_). A node with an energy value smaller than the threshold value will be removed from the CH selection process. Equation (4) is used to measure each node’s threshold power (*n*):
  (4) $$E_{T}\left(n\right)=N_{\mathrm{adjacents}}*q{*E}_{\mathrm{elec}}+N_{\mathrm{adjacents}}*q*\left[E_{\mathrm{elec}}+{\;\beta }_{\mathrm{fsm}}\right]*d^{2}\;$$
  where the node energy threshold is represented by E_T_(*n*), the number of neighbouring nodes is represented by N_adjacents_, the free space model is β_fsm_, and the interval between transmitter and receiver nodes is represented by *d*. Furthermore, each node (n) sends *data_message* with its ID and energy level to all adjacent WSN nodes, which is used to measure and update the adjacent WSN nodes table for each loop.
3. In each cluster, the mean distance (*β*) between candidate CH and its neighboring WSN nodes is important. If it is smaller, then the chances of selecting that nominated CH are higher. Equation (5) is used to calculate the value of *β*:
  (5) $$\beta \left(n\right)=\frac{\sum_{j=1,\;j\neq n}^{Ω}d_{n⇢j}}{Ω}\;$$
  where Ω is the number of WSN nodes located in each cluster. Moreover, Equation (6) can be used to work out the distance threshold for each WSN node:
  (6) $$d_{T}=\left(\sqrt{\frac{\beta_{\mathrm{fsm}}}{\beta_{\mathrm{trm}}}}\right)\;$$
4. The shortest path to AP (*d_A_*) must be calculated. The Received Signal Strength Indicator (RSSI) between WSN nodes and the AP can be used to measure the *d_A_* value. The AP can also use GPS to calculate the positions of all WSN nodes [61]. The ideal CH is evaluated using these parameters, depending on the highest weight value measured using (7):
  (7) $$d_{T}=\left(\omega =\frac{Ω*\;2E*\beta }{d_{A}}\right)\;$$

According to (7), the ideal value of CH has the highest energy, is located near the AP location, and is located at the middle of the other WSN nodes in each cluster. Moreover, we tried to give the residual energy parameter a higher score than the rest of the parameters due to its importance. Therefore, in this case, the consumption of communication energy between the WSN nodes and their CH, as well as between the CH and the AP, will be the lowest.

In this study, we use the AP to pick the CHs from the nodes deployed in the AP area. This collection is allocated to the numerous zoning regions covering the AP, after which CH_Rotations will be self-organized. The CH then sends the *adv_CH* message, which broadcasts its ID within its own domain. Algorithm 1 has a detailed discussion of CH_Rotations in the new LEACH protocol in each zone area.

**Algorithm 1** CH_Rotations1. Set *E_T_*
2. **for** each loop **do**
3.  **for**
*each n*
∊(1,N)
**do**
4.   *Find E*
5. **   if**
*(E ≤ E_T_)*
**then**
6.    *Calcuate d*
7.    *Calcuate d_A_*
8.    *Calucate β*
9.    *Compute ω*
10. **   end if//**line 5
11. **  end for*/***/line 2
12.  *A new CH is selected based on the highest ω*
13.  *A new CH sends Adv-CH messages*
14.  *The N nodes send Join_Req to new CH*
15. **end for**

The algorithm always starts by counting the loop and then in each cluster will search for the best CH to cluster group through computing *ω* for each WSN node.

### 3.2. Water Cycle Detection Approach

One of the essential aims of this study is to introduce the Water Cycle (WC) algorithm as a feature selection technology to identify the least number of attributes and achieve better accuracy in machine learning algorithms, as well as to reduce predictable features and evaluate the recommended extraction over the benchmark dataset along with the actual dataset. Similar to meta-heuristic algorithms, the recommended approach begins with an initial population named raindrops in which the best individual is chosen as a sea. Subsequently, several good raindrops “features” are selected as a river whereas the other raindrops are considered streams that flow into the sea as well as rivers. Therefore, on the basis of its volume flow, water is taken from riverbeds. Moreover, the amount of water in streams entering the sea or rivers varies from one stream to another. The rivers that flow into the sea are the hilliest sites [53,62].

**Figure 3:**
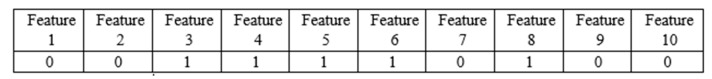
Representation of selected features.

The WC method utilizes several representations to code the entire *F* of the feature in a vector of length *m*, where *m* represents the number of features. Each portion of such a vector contains a label indicating whether the features are dropped or chosen. Figure 3 depicts an example of how solutions can be represented. In this case, 6 features (3, 4, 5, 6, and 8) are chosen while the others (1, 2, 7, 9, and 10) are dropped.

#### 3.2.1. Initial Features Development

The values of the dataset features are considered as an array. In the optimization terminologies of the practical swarm and the genetic algorithm, the array is called “Particle Position” or “Chromosome” respectively. Therefore, in the recommended approach, the label is “Raindrop Features” for an individual feature. In the *M_var_* dimensional feature selection problem, the raindrop represents an array of *1 × M_var_*. This array is illustrated as follows:

(8) $$Feature\;of\;Raindrop=\left[x_{1},x_{2},x_{3},\ldots .,x_{M}\right]\;$$

At the beginning of the feature selection, a candidate is represented by a *M_pop_ × M_var_* raindrop size array (i.e., raindrop features). Thus, the random matrix *x* is provided as (columns and rows that make up the design variable quantity plus feature selection quantity):

(9) $$Feature\;Raindrops=\left[\begin{array}{c} Raindrop_{1}\\ Raindrop_{2}\\ Raindrop_{3}\\ \vdots \\ Raindrop_{M_{pop}} \end{array}\right]*\left[\begin{array}{ccc} x_{1}^{1}x_{2}^{1}x_{3}^{1} & \cdots & x_{M_{var}}^{1}\\ \vdots & \ddots & \vdots \\ x_{1}^{M_{pop}}x_{2}^{M_{pop}}x_{3}^{M_{pop}} & \cdots & x_{M_{var}}^{M_{pop}} \end{array}\right]$$

Each value of the decision variable $\left(x_{1},\;x_{2},\;x_{3}\ldots \;x_{M_{var}}\right)\;$ can be described as the following numbers (0 or 1), where *M_vars_* and *M_pop_* are the number of design variables as well as the number of raindrops (preliminary feature selection), respectively. Further *M_pop_* raindrops are generated, thus the raindrop cost is achieved by evaluating the cost function (Cost) as follows:

(10) $$Cost_{i}=f\;\left(x_{1}^{i},\;x_{2}^{i},\ldots x_{M_{var}}^{i}\right),\;i=1,2,3,\ldots .,M_{pop}$$

#### 3.2.2. Cost of Solutions

All possible solutions are evaluated according to the fitness selection procedure along with classifier algorithms, namely KNN [63], DT [64], SVM [65], DL [21], and NB [59], to obtain the highest performance accuracy among the classification algorithms and features selected for each solution. For the purpose of maintaining an adequate balance between all the selected features in each of the minimum solutions and providing maximum accuracy for feature selection, the fitness function, i.e., objective function in (11) is used in the WC technique to evaluate solutions in *M_pop_*:

(11) $$fitness=\Phi \gamma_{R}\;\left(D\right)+\partial \;\frac{\left|R\right|}{\left|M\right|}$$

where *γ_R_ (D)* is the rating of classification error for a given classifier; |R| is the total items in the selected subset, |M| is the total number of features in the dataset, *∂* and *Φ* are two parameters that represent the importance of the classification quality and subset length, *∂* ∈ [0, 1] and *Φ* = (*1* − *α*) [50,66].

Many *M_sr_* are selected from among the best individuals (minimum values) of the sea and rivers. The raindrop of the lowest value represents the sea. As a matter of fact, *M_sr_* represents the total quantity of rivers (i.e., the user-defined parameters) plus the individual sea as shown in (12). The remaining preliminary features (raindrops from the streams flowing to the rivers or directly to the sea) are calculated on the basis of (13).

(12) $$M_{sr}=Number\;of\;Rivers+\left(Sea=1\right)\;$$

(13) $$M_{Raindrops}=M_{pop}-M_{sr}$$

To facilitate the allocation of raindrops for sea and rivers in terms of the flow density, this Equation is used:

(14) $$M_{sn}=round\left\{\left|\frac{Cost_{m}}{\sum_{i=1}^{M_{sr}}Cost_{i}}\right|\times M_{Raindrops}\right\},\;m=1,\;2,\ldots ,\;M_{sr}$$

where *M_Sr_* represents the quantity of stream flowing into a given sea or river [53].

#### 3.2.3. Stream Flow to Rivers or Sea

The streams of raindrops are generated and communicate with each other to generate new rivers. In fact, a number of streams might flow directly into the sea. All streams, as well as rivers, end up in the sea (best-chosen features). To illustrate, a stream moves towards a river which lies along a line linking them using a randomly defined distance which can be illustrated as follows:

(15) Xϵ (0,C×di),C>1 

*C* represents a value between 1 and 2 (closer to 2). The best value for *C* might be selected as 2. The existing distance between the river and stream is described as *di*. The *X* value in (15) matches a number that is randomly distributed between 0 and (*C × di*). When the *C* value is greater than 1, it enables streams to run towards the rivers. This idea might also be utilized on the rivers that reach the sea. Therefore, the new location for rivers and streams might be represented as:

(16) $$X_{Stream}^{i+1}=X_{Stream}^{i}+rand\times C\times \left(X_{River}^{i}-X_{Stream}^{i}\right)\;$$

(17) $$X_{River}^{i+1}=X_{River}^{i}+rand\times C\times \left(X_{Sea}^{i}-X_{River}^{i}\right)$$

The *rand* represents a uniform number that is randomly assigned between the values of 0 and 1. Moreover, if the precision provided by the stream works better than the river connecting it, the position of the stream and the river are swapped (i.e., the stream becomes a river and vice versa). This exchange can also occur for the sea and rivers [53].

#### 3.2.4. Evaporation Condition

Evaporation is one of the most important factors preventing the algorithm from rapid convergence (immature convergence) [53]. Generally, water evaporates from lakes and rivers while trees absorb and then release water via photosynthesis. The evaporated water rises to the atmosphere to form clouds, which in turn condense into rain under colder conditions, releasing water to the ground. Accordingly, the rain generates new streams that reach the rivers and these rivers also flow to the sea [67]. In the proposed approach, the evaporation process allows seawater to evaporate as streams/rivers flow into the sea. The following pseudo-code (18) shows how to determine if a river extends into the sea or not:

(18) $$\begin{array}{c} \;If\\ \;\left(\left|X_{Sea}^{i}-X_{River}^{i}\right|<di_{max}\right),\;i=1,\;2,\;3,\ldots ,M_{sr}-1\\ Evaporation\;and\;raining\;process\\ End \end{array}$$

where *di_max_* represents a tiny number (near 0). If the area between a sea and river is smaller than a *di_max_*, this indicates that the river joined the sea. In this case, the process of evaporation is used, and as a result of widespread evaporation, precipitation (rain) begins. A large *di_max_* reduces the search while the small value stimulates the search intensity near the sea. As such, *di_max_* controls the search intensity near the sea (best possible solution). Therefore, the *di_max_* value decreases flexibly as shown below:

(19) $$di_{max}^{i+1}=di_{max}^{i}-\frac{di_{max}^{i}}{\max iteration}\;$$

#### 3.2.5. Raining Process

After the evaporation process is achieved, the precipitation process takes place. In the process of rain, new raindrops form streams at different locations (working equally with the mutation factor of the genetic algorithm). For the purpose of determining the positions of the newly produced streams, this Equation is used:

(20) $$\;X_{Stream}^{new}=LB+rand\times \left(UB-LB\right)\;$$

where *UB* and *LB* are the upper and lower limits defined by the problem investigated, respectively.

Moreover, the best recently formed raindrop is considered a river flowing into the sea. The remaining new raindrops are thought to generate some new streams that flow into rivers or directly into the sea. In order to enhance the convergence rate, as well as the computational performance of the algorithm for the specific problems, Equation (19) is only used for flows that take place directly to the sea. Equation (21) aims to encourage the establishment of watercourses heading directly to the sea and to promote searches near the sea (optimal features) in the potential area of specific problems [67]:

(21) $$X_{\mathrm{Stream}}^{\mathrm{new}}=X_{\mathrm{sea}}+\sqrt{\mu }\times rand\;\left(1,\;M_{var}\right)$$

where *μ* is the parameter indicating the extent to which the near-sea area is surveyed, and *Rand* is the naturally distributed random number. A greater value of *μ* increases the potential to exit the region, while a smaller value of *μ* causes the algorithm to explore in a smaller near-sea area. There is a suitable value for *μ* at 0.1. The term $\sqrt{\mu }$ in (21) mathematically describes the standard deviation. Hence, *μ* defines the concept of variance. Depending on such concepts, individual raindrops created with variance *μ* are assigned the best choice of features achieved (sea) [53].

#### 3.2.6. Convergence Criteria

The water cycle stops when a feature is selected, which occurs when the standard-fit does not change at a predetermined value *Ɛ* = *di_max_* after several iterations or achieving the largest number of generations.

The WC is illustrated along with the classifier algorithms for a WSN-DS dataset in Algorithm 2.

**Algorithm 2** WC Feature Selection method1. *Set Mpop, Msr, dimax, Max_Iteration.*
2*. Execute Equations (11) and (12)//*to determine the number of streams flowing into rivers and sea
3*. k = Features (WSN-DS)*
4*. Mpop (1:k) = random initial population*
5*. Set the classifier algorithm (DT, KNN, SVM, DL, NB)*
6*. Calculate the fitness function for Mpop (1:k) using Equation (10)*
7*. Sort fitness values in descending order*
8*. F_sea = best fitness for initial populations*
9*. F_river = best next fitness after the F_sea*
10*. F_stream = Execute Equation (13)/*/to determine the number of streams flow to their corresponding rivers and sea, which is considered the best fitness after F_river
11*. t = 0*
12*. **while** (t < Max_Iterration) **do***
13*. **for** i = 1: Mpop do*
14*.   new_stream = Execute Equations (15) and (16)//*to find new stream flows
15*.    F_new_stream = Execute Equation (10) for new_stream*
16*. **  if** F_new_stream < F_river **then***
17*.    River = new_stream*
18*. **  if** F_new_stream < F_sea **then***
19*.    Sea = new_stream*
20*. **  end if**/*/line 16
21*. **  end if**/*/line 18
22*. **  **new_river = Execute Equation (17)/*/to find new river flows
23*.    F_new_river = Execute Equation (10) for new_river*
24*. **  **if F_new_river < F_sea then*
25*.    Sea = new_river*
26**.**
***   end if****/*/line 24
27*. **  end for**/*/line 13
*28. ** for** i = 1: Msr **do***
29*. **  if** (distance (Sea and River) < dimax) or (rand < 0.1) **then***
30*.   New_stream = Execute Equation (18)*
31*. **  end if**/*/line 29
32*. ** end for**/*/line 28
33*. t = t + 1*
34*. **end while***
35*. print the performance accuracy and number of selected features*

The WC can be integrated with various machine learning classification algorithms such as DT, SVM, KNN, DL classifier, and Naive Bayes (NB). The integrated scheme that has the best performance metrics will be determined to be a WC approach in the CH node in the WSN simulation. Furthermore, the WC technique is benchmarked with various feature selection techniques such as PSO, SA, HS, and GA on the same classifier algorithms and same dataset. Based on [62], WC has been demonstrated to be a highly efficient statistical algorithm compared to many other feature selection techniques and has demonstrated superiority over them. This is in addition to the accuracy of the algorithm in terms of the number of evaluation functions for each problem. It has also been empirically proven that WC can offer competitive solutions compared to most metaheuristics. Therefore, since the WSN traffic data is statistical, it is expected to give the best results.

### 3.3. Decision Making

**Figure 4:**
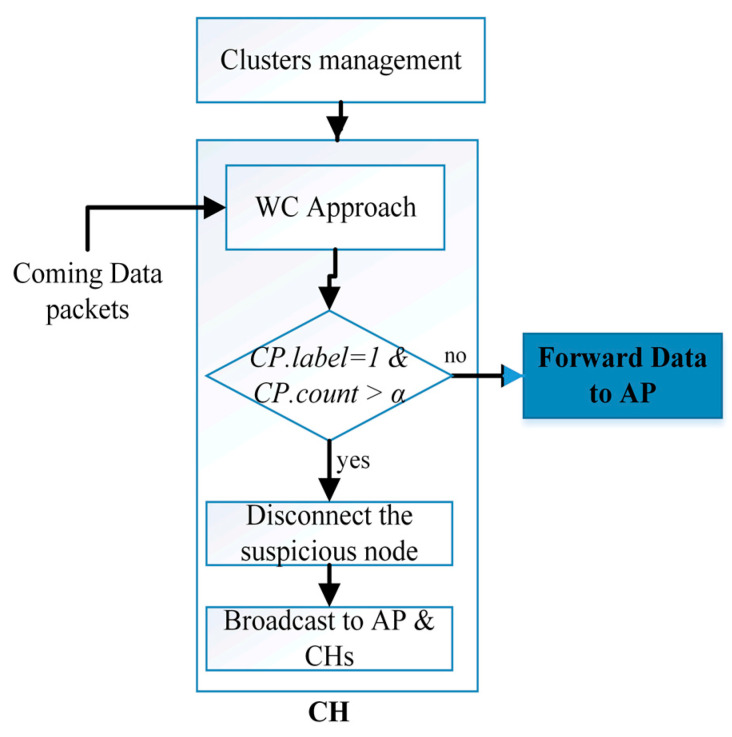
Decision making process in each CH.

At this stage of our proposal system, the CH monitors the number of duplicate suspicious packets; if there is a confirmation of duplication, the CH node will cut off the connection with the suspicious WSN node, add WSN node information to its blacklist, and send a broadcast message to the all neighbor CHs and the AP informing them about the suspicious WSN node. This process is illustrated in Figure 4.

Based on Figure 4, each data Packet that Comes (CP) will be scanned; if it is labelled as 1, CH will count it as a suspicious packet and then count the number of times it repeats over a specified period of time. Subsequently, if the *CP.count* exceeds *α,* the CH will execute the broadcast command and disconnect the connection, where α is the maximum number of occurrences of suspicious packets. This process gives an opportunity to reduce the false-negative rate in our proposal by confirming the attack. As for the values of time and α, they are subordinate to the policy of the organization or company, which will determine the number of repeated DoS or DDoS packets in a period of time to be considered malicious attacks or not.

## 4. Data Collection

In this work, we tested our proposed method using a WSN traffic dataset associated with DoS and DDoS attacks. There are different types of network traffic datasets such as CICDDoS2019 [8], BoT-IoT [68], and WSN-DS [29]. The CICDDoS2019 and BoT-IoT datasets were collected from various device types (servers, sensors, routers, and switches) that were originally classified as IoT networks and not WSNs, whereas the WSN-DS was collected from WSNs by using the LEACH protocol. The Label feature in WSN-DS data categorizes different types of DoS attacks (Blackhole, Grayhole, Flooding, and Scheduling). Moreover, it contains 18 features with approximately 325,000 records. The features disclosed are: WSN node ID, occurrence time, whether the WSN node is CH, identity of the CH for WSN nodes that are not CH, distance between WSN node and CH, advertisement messages sent from CH to WSN nodes, advertisement messages received from CHs, join request messages sent from WSN nodes to CH, join request messages received by CH from WSN nodes, TDMA advertisement messages sent to WSN nodes from CH, TDMA advertisement messages received by CHs from WSN nodes, rank, number of data packets sent from WSN nodes to CH, number of data packets received from CH, number of data packets sent to the AP, distance between CH and AP, send code, and label. These features and their sequence are illustrated in Table 3.

As with our goals to reduce node power consumption and move the defense decision to edge AP, we converted these four types of DoS attacks into one class labelled “1”; the label “0” indicates normal traffic. The amount of attack data is 88.6% less than that of normal data. Furthermore, the attack data is distributed as 33% Blackhole, 11% Flooding, 35% Grayhole, and 21% TDMA.

## 5. Implementation and Evaluation

In this section, we first discuss the analysis of the WC approach using the WSN-DS traffic dataset. The simulation environment and experimental performance of the WC approach are discussed, and then an analysis of the results for this approach is presented. After that, another environment was applied based on the output of the first analysis to show the impact of this approach on the lifetime of the WSN.

### 5.1. Complexity Analysis for the Water Cycle Detection Approach

In this work, the accuracy performance metric was utilized to analyse the WC detection approach using the WSN-SD Dataset in different classification categories. The aims were to find the highest number of True Positives (*TP*) and True Negatives (*TN*) and the lowest number of False Negatives (*FN*) and False Positives (*FP*). The number of *TN* indicates that valid traffic is recognized, while *TP* is the likelihood of irregular traffic being recognized. The number of *FN* illustrates the likelihood of attack flows being identified as normal flows, while *FP* represents the likelihood of normal flows identified as attack flows. The False Positive Rate (FPR) indicates the wrongly defined attack ratio and the inverse False Negative Rate (FNR) represents the cumulative sum of incorrect forecasts. Accuracy is the percentage of accurate model prediction for all types of predictions produced. The following Equation represents the accuracy performance metric used in this analysis:

(22) $$Accuracy=\frac{TP+TN}{TP+TN+FP+FN}$$

#### 5.1.1. Water Cycle Parameter Settings

We investigated the evolution of WC solutions under several parameter settings. These parameters were chosen for the WC scheme because the WSN-DS dataset differs from other datasets, and we needed to map the best results according to the fitness function mentioned earlier in (11). The *di_max_* was set as 1E03. The most important parameters are *M_sr_* and *M_pop_*. To clarify, *M_sr_* represents the total number of rivers (i.e., parameters defined by the user) plus the individual sea, while *M_pop_* represents the number of raindrops (i.e., the preliminary population of features). Consequently, this section highlights the impact of changes in individual parameters. Table 4 examines three contrasting scenarios (M_pop_ = 2, 4, and 8).

Experimental research showed that the specific relationship between *M_sr_*, *M_pop_* and the number of features provides the best results. In each scenario examined, the highest number of iterations was set at 100 for each run. The best result was selected based on the fitness function value. The best scenario was the seventh one, which had *M_pop_* = 8 and *M_sr_* = 3. Regarding other benchmark feature selection methods, we set their parameters as shown in Table 5. The rest of their parameters remained the defaults. In addition, for all methods, we defined a population size of eight and a maximum iteration limit of 100.

#### 5.1.2. Evaluation of the Water Cycle Approach

WC was used with the WSN-DS dataset to detect the most important features. Moreover, five classifier algorithms were evaluated in this work along with the WC technique. The standard cross-validation was used, which requires the training and validation sets to crossover in successive rounds. All this means that every data point can have a chance to be validated. A sub-category of cross-validation was *k*-fold data cross-validation. After using this cross-validation, the data were split into *k* segments of training and testing, which were either equal or assumed to be approximately equal in size or folds. As such, *k* iterations were performed to practice and train results alongside validation in such a way that a different data fold was maintained within each iteration for validation, while the (*k* − 1) iterations were utilized for basic learning. In the context of data extraction, text mining, and machine learning, (k = 10) 10-fold cross-validated appears to be the most widely used and widespread value for the data [62]. As such, we first used 90% training and 10% testing, then 80% training and 20% testing and so on until reaching 10% training and 90% testing, after which we took the average.

At this point, it must be emphasized that the results shown in the rest of this section are based on average scores for more than 20 algorithms (combining feature selection methods and classifier algorithms). In order to facilitate the computation of the average in the results of the implementation of the algorithm, we repeated the execution of the algorithms about 100 times for each execution.

To begin with, we reviewed the accuracy performance of the classifier algorithms, namely DT, SVM, KNN, DL, and NB, on the WSN-DS dataset, along with the total number of dataset features. Table 6 shows the accuracy results for these classifier algorithms.

According to the values retrieved from the Table, the DT classifier displayed the highest accuracy result with a rate of 99.6%. The SVM was 98.8%, KNN was 98.5%, DL was 97.7%, and NB was 56.9%. We note that the NB showed the worst performance in terms of accuracy. One of the reasons for DT’s good results is that the type of data collected from network traffic in most of its features are numerical statistical values [68]. Therefore, statistical and logical machine learning techniques provide good results with less training time.

In order to illustrate the effect of feature selection techniques on the accuracy of detection using the WSN-DS dataset, we integrated machine learning classifier algorithms along with feature selection techniques as mentioned previously to detect the most important features of the WSN-DS dataset. The WC technique was benchmarked with various feature selection techniques such as PSO [21], SA [22], HS [19], and GA [23] using the same classifier algorithms and dataset. The output of these operations is shown in Table 7. The Table also shows the accuracy performance of each integrated technique, number of WSN-DS features they used, and sequence of the features.

Based on the results of Table 7, we found that the best accuracy performance was achieved with the WC feature selection technique. It gave accuracy results of approximately 100% when using the DT and DL classifier algorithms. Moreover, if we take into account the average accuracy performance for each feature selection technique, WC continued to have the best accuracy performance, followed by POS, HS, GA, and SA in that order. With regard to the classifier algorithms used with the WC feature selection technique, we found that the DT and DL algorithms were equally the best in terms of performance accuracy and number of identified WSN-DS features. The accuracy performance metric was 100% for both and the number of WSN-DS features identified was 1. The WSN-DS feature sequence was 17 for both classifiers. The accuracy performance of the remaining classifier algorithms was distributed as SVM 99.04%, KNN 98.9%, and NB 81.98%. The number of selected WSN-DS features was also distributed between 12 and 15.

Due to the similarity of performance accuracy and number of selected features between the WC + DT and WC + DL, we resorted to using Friedman and Iman–Davenport statistical tests [69] in order to compare the two types. The Friedman and Iman–Davenport tests are designed to demonstrate whether there is a statistical difference between classes (crossover operators) [70]. Table 8 shows the average ranking of the WC with machine learning algorithms according to Friedman’s test (the lower the value, the higher the rank). The last two rows in Table 8 refer to the p-value of the Friedman and Iman–Davenport statistical tests [69]. The results tabulated in Table 8 show that the WC+DT had the lowest value, so it was rated first.

**Figure 5:**
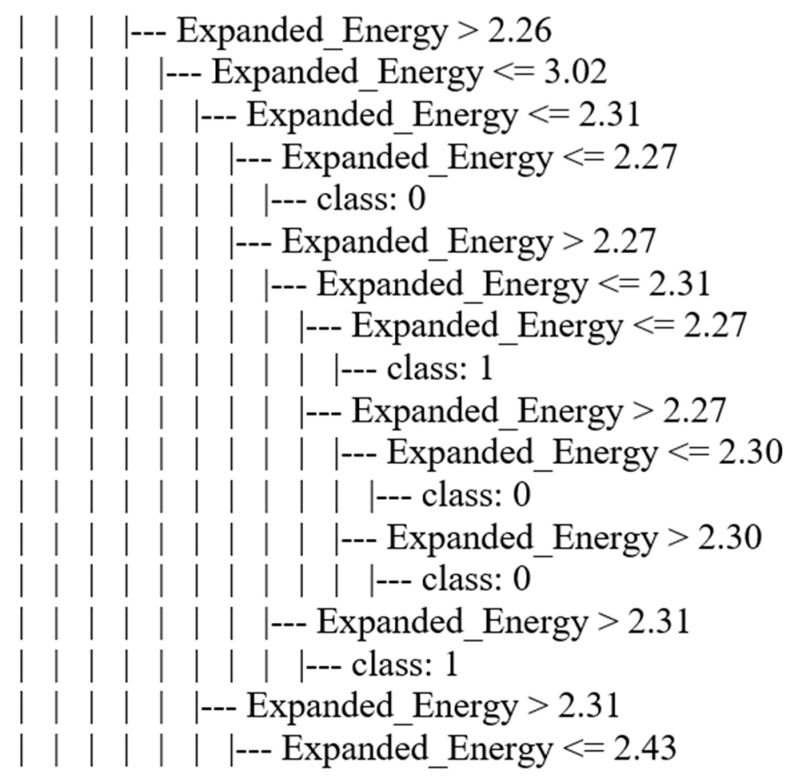
A sample of the WC+DT output model for the WSN-DS dataset.

We chose the WC + DT technique to implement in a WSN simulation. We selected it based on the highest performance in accuracy and Friedman’s test ranking. To implement this technique in WSN simulations, we needed to determine the WC+DT output model from the training and testing processes. Therefore, the best value for maximum depth was set as 10 based on the accuracy performance metric. The dependent variable (Label feature) of the WSN-DS dataset had two values (0 “Normal” or 1 “Attack”), and thus the portion of the WC + DT output model from the WSN-DS dataset represented the relationship between the independent variables and dependent variables as illustrated in Figure 5.

### 5.2. Complexity Analysis for the Lifetime of WSNs in CH_Rotations and WC + DT Approaches

#### 5.2.1. Simulation Environment

Contiki operating system with Cooja simulator were used to simulate WSN architecture [71]. The modified LEACH protocol was used as a clustering management subsection to manage and control the WSN node’s hardware and software. The simulation was run on a machine with a 1.8 GHz Intel Core i5 processor, 6 MB cache, and 8 GB RAM. The default parameters used in the architecture of the wireless network are plotted in Table 9, and parameter values in the table are taken from the values in [72].

In the simulation, the WSN nodes were initially spread across dimensions of 500 × 500 terrain associated with nine CHs at initial values distributed in different sub-regions within the AP coverage region. The initial energy of the WSN node and the number of used WSN nodes were chosen based on the proposed simulated need, which we will discuss later in detail. Moreover, each ordinary node sent 64 packets per second to its CH node and each packet size was 1000 bits. If the node was CH, the received packets were forwarded to AP.

#### 5.2.2. Experimental Metrics and Results

In this step, the CH_Rotations algorithm was first analysed to show its effect on WSN lifetime. Next, we analysed the impact of the WC + DT technique incorporating the CH_Rotations algorithm on WSN lifetime. The lifetime of the network was calculated when the power of some WSN nodes reached 0.

**Figure 6:**
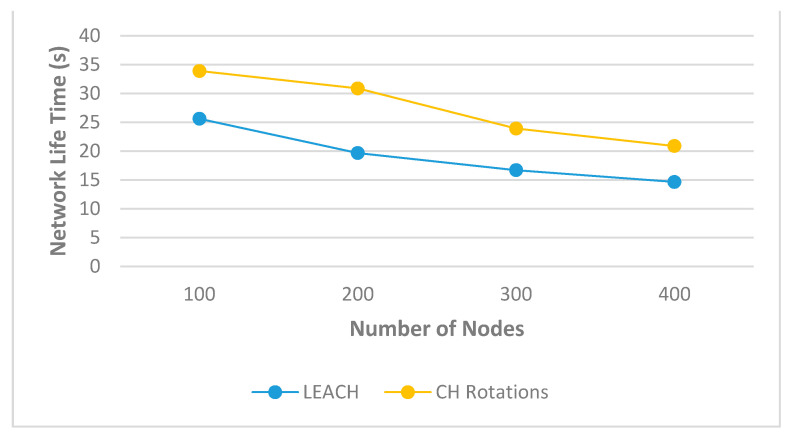
Analysis of the effect of WSN nodes number on the network lifetime.

With regard to the evaluation of the “CH_Rotations” proposal scheme, we set the initial energy of the WSN node to 1 joule and the simulation time to 200 s to allow some of the WSN nodes’ energy to reach 0. The number of nodes was increased by 100 each time and the number of CHs was set to 9 CHs for both schemes (CH_Rotations and LEACH). Analysis of the effect of WSN node number to network lifetime for both schemes is depicted in Figure 6.

As illustrated in Figure 6, an increase in the number of WSN nodes resulted in reduced network lifetime when using either technique. The reason behind this decrease is the effect of over-connecting ordinary WSN nodes to the CHs. However, the CH_Rotations algorithm showed an improvement in network lifetime compared to the LEACH technique, because the process of selecting CH was done mathematically based on several factors and not randomly as in LEACH. In addition, based on the effect of distance and received and transmitted signal strength between the WSN nodes, an increase in distance increases and decrease in signal strength correlates to an increase in energy consumption and a decrease in network transmission rate. Thus, selecting CH positions close to neighbouring WSN nodes and to the AP provides good communication and conserves the network lifetime. Finally, the result showed that CH_Rotations improved network lifetime by 24%, 36%, 30%, and 29% compared to the LEACH technique when using 100, 200, 300, and 400 WSN nodes, respectively.

For DoS detection evaluation using the WC + DT technique, we simulated it with the CH_Rotations algorithm and ran it to see the effect of monitoring and packet inspection in each CH on the network lifetime. The WC + DT output model was distributed to all WSN nodes, and when a WSN node became a CH, it started monitoring the consumption energy, then found expanded energy and calculated *y* (Label feature) for each packet. For each positive *y* (Label = 1), the CH created a counter table for the WSN node that sent suspicious packets, and calculated from one to α; if the counter reached this value in a period of time (*t*), the CH blocked this node and sent a broadcast message to the AP for this case. Moreover, these counter tables were forwarded between WSN nodes so that all of them were aware of these numbers. The idea of a counter table is important and is meant to reduce the FNR in WSNs.

**Figure 7:**
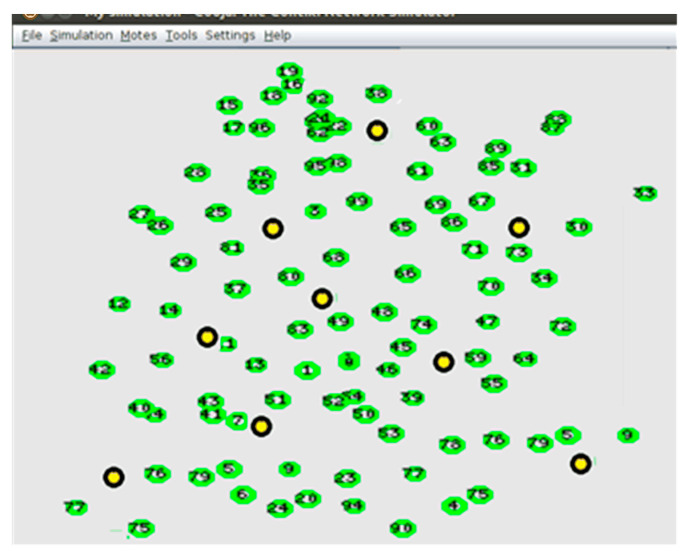
Simulation WSN nodes.

In the WC+DT technique simulation, the WSN nodes were initially spread randomly as seen in Figure 7. The initial energy of the WSN node was set to 1.5 Joules and the simulation time to 500 s to allow some of the WSN nodes’ energy in the first scenario to reach zero.

**Figure 8:**
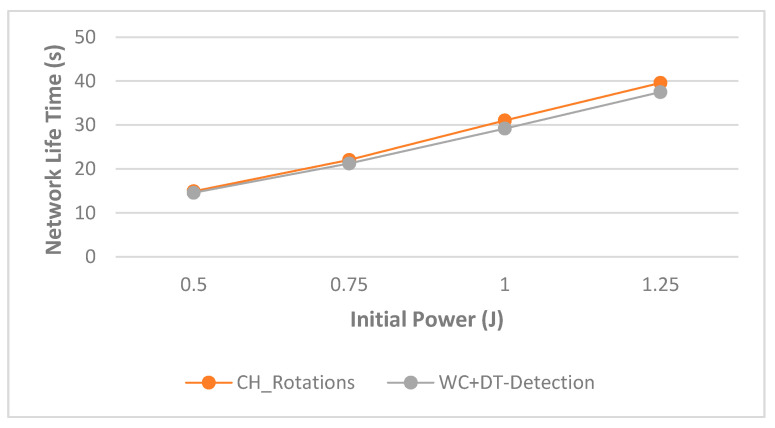
Analysis of the effect of WC+DT technique on WSN lifetime.

The monitoring of nodes’ packet activity occurred in a constant time interval, and the statistical calculation for each DoS attack during these time intervals (*t*) was the same data features calculation of [29]. Regarding the WC+DT detection model, we supposed that the energy consumption per each packet inspection would be 0.001 J, and depending on this energy consumption value, the analysis effect of the WC+DT DoS detection on the WSN lifetime is illustrated in Figure 8. As illustrated in Figure 8, the increments of initial power in WSN nodes increased the lifetime of WSNs in both scenarios. This result is due to the positive relationship between WSN node initial power and time intervals. Moreover, from the same Figure 8, we can observe that the variation in the network lifetime between two scenarios increased with increases in the initial power of the WSN nodes.

This variation increased from 2% to 6% when the WSN node initial power increased from 0.5 to 1.25 J. The reason for the increase in this variance was due to the increase in the rate of packets received by the CH nodes, which in turn led to an increase in the rate of inspection and verification messages. This in turn led to an increase in the rate of power consumption within the CH nodes, and thus the result was a decrease in the network lifetime. The results show that the WC+DT detection algorithm decreased the network lifetime by 2%, 4%, 6%, and 6% compared to the WC+DT-free scenario for WSN node initial power of 0.5, 0.75, 1, and 1.25 J, respectively.

## 6. Conclusions and Future Work

Network traffic is becoming more complex due to the increase in the amount of data transferred between WSN nodes resulting from increased usage. It is important to reduce power consumption and improve data protection in these networks, especially in order to prevent DoS attacks. In this paper, we modified the LEACH clustering protocol to improve its performance by adding various factors such as WSN node residual power, distance between WSN nodes, and the distance between the candidate CHs and the AP. Moreover, we analysed the performance of various feature selection techniques along with different machine learning algorithms to improve DoS detection in the WSN-DS dataset. The feature selection techniques used were WC, SPO, HS, and GA, and with each feature selection technique different machine learning algorithms such as DT, DL, KNN, NB, and SVM were used. Performance accuracy metrics were used to evaluate each algorithm. The best technique for feature selection was WC as its average performance accuracy was 2%, 5%, 3%, and 3% higher than that of PSO, SA, HS, and GA, respectively. The best machine learning algorithm results when used with WC were displayed by the DT and DL algorithms, which had the highest accuracy of 100% and the lowest number of features (Expanded Energy). The rest of machine learning algorithms achieved accuracy performances of SVM 99%, KNN 99%, and NB 81% with different numbers of WSN_DS features distributed between 12 and 15. The Friedman and Iman–Davenport statistical tests were used to select which of the two highest-performing machine learning algorithms (DT or DL) was most appropriate. The WC+DT had the lowest score of 5.449, hence WC + DT was selected as the best DoS detection technique.

Furthermore, Cooja simulator software was also used to obtain WSN lifetime. The simulation environment was managed by either CH_Rotations, a modified LEACH protocol, or the LEACH standard protocol. CH_Rotations improved the WSN lifetime by 30% compared to the standard LEACH routing protocol. The WC + DT technique consumed 5% of the total WSN lifetime compared to the WC + DT-free scenario. In future, we plan to collect a new WSN dataset from the 6LoWPAN protocol and add new features such as packet size per stream, dropped packets per stream, flow change ratio, and packet change ratio.

## Figures and Tables

**Table 1:** Taxonomy of WSN DDoS attacks.

WSN Layers	Description
Perception layer	Jamming Tempering Scheduling
Perception MAC Layer	Collision Exhaustion
Network or Routing Layer	Blackhole Grayhole Hello
Transport Layer	Flooding
Application Layer	Overwhelming nodes Path-Based DoS

**Table 2:** Taxonomy of different machine learning algorithms in detection attacks.

Category	Reference	Technique	Dataset	Accuracy	Goals	Limitation
Statistical-based	[15]	Game theory and an autoregressive model	Live format simulator (Matlab)	81%	Reduce detection power consumption in the intrusion detection process	Accuracy is low
MLP	[23]	Sequential feature selection with MLP algorithm	NSL-KDD	99.7%	Reduce DDoS attacks	The proposal is not considered a WSN restriction
Statistical-based	[20]	K-medoid clustering technique	Live format simulator (NS-2)	-	Attacks detection	Accuracy unknown
Deep Learning	[58]	Deep Neural Network	WSN-DS	99%	Improve intrusion detection in IoT networks	The proposal’s consumption makes it unsuitable for WSNs
Deep Learning	[13]	Deep Learning-based Defense Mechanism	Live format network forward packet	90%	Improve DDoS detection in WSNs	No energy consumption tested on WSNs
Statistical-based	[59]	Binary Logistic Regression (BLR)	Live format simulator (Monitoring Tool)	96–100%	Improve DoS detection in WSNs	Data features are few and do not cover the majority of common attacks
Deep Learning	[58]	Deep Neural Network	WSN-DS	99%	Improve intrusion detection in IoT networks	The proposal’s consumption makes it unsuitable for WSNs

**Table 3:** WSN-DS Features Sequence.

WSN-DS Feature	Sequence
Time	1
Is_CH	2
Who-CH	3
Distance to CH	4
ADV_S	5
ADV_R	6
Join_S	7
Join_R	8
SCH_S	9
SCH_R	10
Rank	11
Data_S	12
Data_R	13
Data_sent_to_AP	14
Dist_CH_to_AP	15
Send_code	16
Expanded_energy	17
Label	18

**Table 4:** Some scenarios of parameters for the water cycle as feature selection.

Scenarios	*Mpop*	*Msr*
1	2	3
2	2	5
3	2	9
4	4	3
5	4	5
6	4	9
7	8	3
8	8	5
9	8	9

**Table 5:** Feature selection parameters settings.

Technique	Parameter	Value
GA		
	Mutation rate	0.5
PSO		
	Number of selection	3
	Constant-1	2
	Constant-2	2
HS		
	HRCR	0.7
	PAR max	0.8
	PAR min	0.2
SA		
	Initial Temp	0.2
	Temp reduction rate	0.87

**Table 6:** The accuracy results of five classifier algorithms for WSN-DS dataset.

Classifier Algorithms	Accuracy	#Features
DT	99.5922	18
KNN	98.512259	18
NB	56.87	18
SVM	98.817526	18
DL	97.6987	18

**Table 7:** Results of five classifier algorithms using various feature selection techniques.

Techniques	Accuracy	#Features after Selection	Feature Sequence
WC + DT	100	1	17
WC + SVM	99.0356	15	1, 3, 4, 5, 6, 7, 8, 9, 10, 11, 12, 13, 15, 17, 18
WC + KNN	98.92145	16	1, 3, 4, 5, 6, 7, 8, 9, 10, 11, 12, 13, 14, 15, 16, 18
WC + NB	80.98	12	1, 3, 5, 6, 7, 9, 10, 11, 12, 13, 14, 18
WC + DL	100	1	17
POS + DT	99.4278	10	1, 5, 6, 7, 9, 10, 11, 13, 16, 17
PSO + SVM	98.9156	15	1, 2, 3, 4, 5, 6, 7, 8, 9, 10, 11, 12, 15, 16, 18
POS + KNN	98.6	14	1, 2, 3, 4, 5, 6, 7, 8, 9, 10, 12, 14, 16, 18
POS + NB	77.1	13	1, 2, 4, 5, 6, 7, 8, 9, 10, 13, 15, 16, 18
POS + DL	97.6891	8	1, 4, 5, 6, 8, 10, 15, 16
SA + DT	99.3471	7	2, 3, 5, 7, 10, 15, 17
SA + SVM	98.267	9	1, 2, 4, 6, 7, 9, 10, 15, 16
SA + KNN	98.599	15	1, 2, 3, 4, 5, 6, 7, 8, 9, 10, 11, 13, 15, 16, 18
SA + NB	58.9	14	2, 3, 4, 5, 6, 7, 9, 10, 11, 12, 13, 14, 15, 18
SA + DL	97.6913	7	7, 8, 10, 11, 13, 15, 16
HS + DT	99.3594	8	5, 7, 8, 10, 12, 14, 15, 17
HS + SVM	98.183	10	5, 6, 7, 8, 9, 10, 12, 13, 14, 16
HS + KNN	98.527	13	1, 2, 3, 4, 5, 6, 7, 8, 9, 11, 13, 16, 18
HS + NB	78.6	13	1, 3, 4, 5, 6, 8, 10, 11, 12, 13, 14, 15, 18
HS + DL	89.9296	10	1, 4, 5, 8, 10, 11, 13, 14, 15, 18
GA + DT	99.5794	10	1, 3, 4, 5, 6, 7, 8, 10, 11, 17
GA + SVM	98.1789	13	1, 2, 3, 4, 5, 6, 7, 8, 9, 11, 15, 16, 18
GA + KNN	98.714	12	1, 4, 5, 6, 7, 8, 9, 10, 11, 12, 14, 15
GA + NB	68.92	16	1, 2, 3, 4, 5, 6, 7, 8, 9, 10, 11, 12, 13, 14, 16, 18
GA + DL	97.6993	11	1, 4, 5, 6, 8, 11, 12, 13, 15, 16, 18

**Table 8:** Average ranking of Friedman test for WC with machine learning techniques.

Techniques	Average Ranking
WCA + NB	9.71
WCA + KNN	6.68
WCA + SVM	6.51
WCA + DL	5.99
WC + DT	5.94
Friedman test (*p*-value)	0.00
Iman–Davenport (*p*-value)	0.00

**Table 9:** Simulation parameters used.

Parameter	Value
WSN node size	60 m × 120 m
ER location	X = 30, Y = 90
Number of CHs	Changeable
Number of WSN nodes	100
Simulation time	500
Message size	6400 bits
Control message size	200 bits
Initial energy (Joule)	1
Two-ray ground propagation models	0.0013 PJ/bit/m4
Free space model	10 PJ/bit/m2
Power consumed by transmitter	50 nJ/bit
Transition power	20 nJ/bit
Power consumed by receiver	50 nJ/bit
Distance threshold	87 m
